# Field safety study of VANGUARD®crLyme: A vaccine for the prevention of Lyme disease in dogs

**DOI:** 10.1016/j.jvacx.2020.100080

**Published:** 2020-10-22

**Authors:** Richard T. Marconi, Nicole Honsberger, M. Teresa Winkler, Nikki Sobell, Vickie L. King, Sharon Wappel, Jacquelien Hoevers, Zach Xu, Jason Millership

**Affiliations:** a Department of Microbiology and Immunology, Virginia Commonwealth University Medical Center, Richmond, VA 23298-0678, United States; b Zoetis Inc., 333 Portage Road, Kalamazoo, MI 49007-4931, United States

**Keywords:** Ixodes, Borrelia, Lyme disease, Vaccine, C6, Ticks, Chimeritope, Borreliella

## Abstract

Here we report the results of a large-scale pre-license safety study in which two serials of VANGUARD®crLyme, a vaccine for canine Lyme disease, were tested in its target population (dogs) under the conditions of its intended use. Six-hundred and twenty dogs, from three distinct geographic regions of the United States were enrolled in this study with each receiving two doses of vaccine by subcutaneous injection 3 to 4 weeks apart. Approximately one-third of the dogs were of minimum age (≤8 weeks of age) to meet regulatory requirements. Safety was evaluated by observation of local and systemic reactions for at least 10 days after each vaccination. Abnormal health events (AHEs) occurred at low frequencies and no serious AHEs were observed. The results demonstrated that VANGUARD®crLyme is safe for use in healthy dogs 8 weeks of age or older.

## 1 Introduction

Lyme disease (LD), the most common vector-borne disease of canines and humans in North America [1], is caused by the spirochete *Borreliella burgdorferi* (previously classified as *Borrelia burgdorferi*) [2], [3], [4]. VANGUARD®crLyme (Zoetis) is a multivalent subunit vaccine consisting of a proprietary formulation of a modified outer surface protein A (OspA) and a custom designed outer surface protein C (OspC) derived protein referred to as a chimeritope [5], [6], [7]. Chimeritopes are recombinant proteins consisting of a series of defined linear epitopes derived from different protein variants that are incorporated into a single protein [7]. Chimeritopes can be custom designed to elicit broad antibody (Ab) responses to one or more pathogens. The chimeritope in VANGUARD®crLyme, designated as Ch14, harbors 14 distinct linear epitopes [8] from diverse *B. burgdorferi* OspC types. Vaccination with VANGUARD®crLyme triggers the production of antibodies (Abs) to OspA and OspC [9]. OspA is a tick-phase protein that is not produced in mammals [10]. In unfed ticks, OspC production is low but it is upregulated during the tick feeding process and continues to be produced at high levels during early stage infection in mammals. VANGUARD®crLyme was designed to include OspA and an OspC derived protein with the rationale that antibodies to these proteins can target spirochetes in both ticks and mammals and thus protect against infection through two independent and synergistic mechanisms.

In this report, we present the results of a field safety study. VANGUARD®crLyme was delivered in a two-dose series to 620 dogs. AHEs were minimal and occurred at low frequency. The results demonstrated that VANGUARD®crLyme is safe for use in dogs 8 weeks of age or older. In an accompanying paper by Marconi et al. titled “VANGUARD®crLyme: A Next Generation Lyme Disease Vaccine That Prevents *B. burgdorferi* Infection in Dogs”, efficacy study results are presented.

## 2 Materials and methods

### 2.1 Description of study participants, inclusion and exclusion criteria

A total of 620 pure or mixed breed dogs were enrolled. Only healthy client-owned and purpose-bred dogs were included with no restrictions based on breed, body weight, or vaccine history. Dogs with prior history of immune system disorders, anaphylaxis, angioedema, wheals, and pregnant or lactating dogs were excluded. For client-owned dogs, the owners were required to provide written permission prior to the administration of the vaccine. Of the 620 dogs, 203 were ≤ 8 weeks of age and the remaining 417 were ≥ 9 weeks of age at the time of the first vaccination. The safety study was conducted at 11 veterinary clinics and 2 commercial breeder facilities. Breeder facilities were included in order to meet the USDA requirement that 1/3 of the dogs be ≤ 8 weeks of age. Three study regions were established in the Northeast (Connecticut, Pennsylvania, and New York), Midwest (Michigan, Indiana, and Wisconsin) and the Southeast (North Carolina). The study sites were selected based on the prevalence of LD and the practice of routine vaccination for *B. burgdorferi*. A randomized complete block design with age (minimum age and older) was employed and replicated for each study region. Order of enrollment was the blocking factor and the ‘individual dog’ was the experimental unit.

### 2.2 Vaccination protocols

Study group T01, which consisted of 312 dogs (102 were ≤ 8 weeks and 210 were ≥ 9 weeks of age), received VANGUARD®crLyme serial number A310400B. Study group T02, which consisted of 308 dogs (101 were ≤ 8 weeks and 207 were ≥ 9 weeks of age), received VANGUARD®crLyme serial number A310401. Vaccine was delivered subcutaneously as a 1 mL dose on Day 0 and on Day 21 or 28 (+/- 1 day). IACUC protocols were reviewed and approved by the Zoetis Veterinary Medicine Research and Ethical Review Board (ERB). The protocols were further reviewed by IACUC’s at each commercial breeder site. Physical examinations were preformed prior to each vaccination. Body weight, body temperature, mucosal membranes, skin/coat, cardiac system, digestive system, respiratory system, urinary system, attitude, and overall condition were assessed.

### 2.3 Post-vaccination monitoring

After each vaccination, the dogs were monitored for immediate AHEs (events that occur within 10–20 min) by the examining veterinarian or trained specialist. The dogs were observed for injection pain responses and any abnormal health event (Table 1). Late AHEs (20 min to 10 days post-second vaccination) were monitored by the dog owners or by veterinarians or trained specialists at breeder facilities. Owners were responsible to communicate unusual or unexpected observations to their examining veterinarian. Both immediate and late post-vaccination AHEs were categorized according to standardized low-level terms developed by the Veterinary Dictionary for Drug Regulatory Activities.

**Table 1:** Frequency Distributions of Immediate Abnormal Health Events.

Abnormal Health Events* N = 1231	Overall N (%)	Related to IVP N (%)
Anaphylaxis, Allergic Oedema, Convulsion, Urticaria	0 (0%)	0 (0%)
Emesis, Injection Site Reaction	1 (0.08%)	0 (0%)
Injection Site Self-Trauma, Injection Site Oedema, Lethargy	1 (0.08%)	1 (0.08%)
Injection Site Paraesthesia	5 (0.41%)	3 (0.24%)
Vocalization at Administration	15 (1.22%)	9 (0.73%)

* Where specific AHEs are listed together in the table, the number of events and the percentages for each are identical.

## 3 Results

### 3.1 Frequency distribution of study completion

Of the 620 dogs enrolled, 611 (98.5%) completed the study. When broken down by age, 198 (97.5%) of the dogs that were ≤ 8 weeks of age and 413 (99%) of those that were ≥ 9 weeks of age at the time of the first vaccination completed the study. Of the dogs that received vaccine serial number A310400B or A310401, 98.4% and 98.7% completed the study, respectively. Completion percentage by sex was 97.7% for females and 99.4% for male dogs. Of the nine dogs that did not complete the study, five were dropped due to non-compliance unrelated to abnormal health events (AHEs), two were dropped due to AHEs unrelated to vaccination, and two were withdrawn by the pet owner. In summary, a high completion rate was observed and no dog was removed due to vaccination induced AHEs.

### 3.2 Frequency distribution of Immediate post-vaccination AHEs

Immediate AHE assessments were reported for 1231 of the 1232 vaccinations (a monitoring report was not received for one vaccination). The most frequent immediate AHE at administration was vocalization (crying, barking, or whining) (Table 1). Of the 15 vocalization events, nine (0.73%) were attributed to the delivery of the vaccine. The second most frequent AHE attributed to vaccination was injection site paraesthesia (local skin reaction; three events; 0.24%). Other potential immediate AHEs are reported in Table 2 with the results broken down by first or second vaccination and by age group. Ten and five minor, immediate AHEs considered related to vaccination were reported for the first and second vaccine doses, respectively. Eleven minor AHEs were reported for dogs ≤ 8 weeks of age and four for dogs ≥ 9 weeks of age at the time the first dose was delivered. There was no significance difference in either the immediate or late AHEs that were attributed to the vaccine serial used. In conclusion, the frequency of immediate AHEs was low and no significant events were noted.

**Table 2:** Distributions of Immediate Abnormal Health Events by Vaccinations.

Vaccination	Abnormal Health Events*	Overall N (%)	Related to Vaccine N (%)
**First** **N = 619**	Injection Site Oedema, Lethargy	1 (0.16%)	1 (0.16%)
	Injection Site Paraesthesia	2 (0.32%)	2 (0.32%)
	Injection Site Reaction	1 (0.16%)	0 (0%)
	Vocalization at Administration	9 (1.45%)	6 (0.97%)
**Second** **N = 612**	Emesis	1 (0.16%)	0 (0%)
	Injection Site Paraesthesia	2 (0.32%)	1 (0.16%)
	Injection Site Self-Trauma	1 (0.16%)	1 (0.16%)
	Vocalization at Administration	6 (0.98%)	3 (0.49%)

**Distributions of Late Abnormal Health Events by Vaccinations**			
**First** **N = 620**	Abdominal Pain, Dehydration, Hyperthermia, Muscle Tremor, Retching, Vocalization	1 (0.16%)	1 (0.16%)
	Anorexia	4 (0.65%)	1 (0.16%)
	Dermatitis	2 (0.32%)	1 (0.16%)
	Diarrhea	10 (1.61%)	2 (0.32%)
	Emesis	4 (0.65%)	0 (0%)
	Injection Site Pain	4 (0.65%)	4 (0.65%)
	Injection Site Oedema	23 (3.71%)	23 (3.71%)
	Lameness	1 (0.16%)	0 (0%)
	Lethargy	6 (0.97%)	3 (0.48%)
**Second** **N = 612**	Diarrhea	3 (0.49%)	0 (0%)
	Emesis	4 (0.65%)	0 (0%)
	Injection Site Oedema	24 (3.92%)	24 (3.92%)

**Distributions of Immediate Abnormal Health Events by Age Group**			
**Age Group**	**Abnormal Health Events**	**Overall N (%)**	**Related to IVP N (%)**
**≤8 Weeks** **N = 401**	Injection Site Paraesthesia	4 (1.00%)	3 (0.75%)
	Injection Site Self-Trauma, Lethargy	1 (0.25%)	1 (0.25%)
	Vocalization at Administration	7 (1.75%)	6 (1.50%)
**≥9 Weeks** **N = 830**	Emesis, Injection Site Paraesthesia, Injection Site Reaction	1 (0.12%)	0 (0%)
	Injection Site Oedema	1 (0.12%)	1 (0.12%)
	Vocalization at Administration	8 (0.96%)	3 (0.36%)

**Distributions of Late Abnormal Health Events by Age Group**			
**≤8 Weeks** **N = 401**	Abdominal Pain, Dehydration, Hyperthermia, Muscle Tremor, Retching, Vocalization	1 (0.25%)	1 (0.25%)
	Anorexia, Lethargy, Dermatitis	2 (0.50%)	1 (0.25%)
	Diarrhea	9 (2.24%)	1 (0.25%)
	Emesis	3 (0.75%)	0 (0%)
	Injection Site Pain	2 (0.50%)	2 (0.50%)
	Injection Site Oedema	28 (6.98%)	28 (6.98%)
**≥9 Weeks** **N = 831**	Anorexia	2 (0.24%)	0 (0%)
	Diarrhea	3 (0.36%)	1 (0.12%)
	Emesis	5 (0.60%)	0 (0%)
	Injection Site Oedema	12 (1.44%)	12 (1.44%)
	Injection Site Pain	2 (0.24%)	2 (0.24%)
	Lameness	1 (0.12%)	0 (0%)
	Lethargy	4 (0.48%)	2 (0.24%)

* Where specific AHEs are listed together in the table, the number of events and the percentages for each are identical.

### 3.3 Frequency distributions of late post-vaccination AHEs

Late AHE assessments were reported for 1232 of the vaccinations (100%). The most frequent late AHE following vaccine administration was oedema (fluid in tissue) at the injection site (40 events; 3.25%). Injection site pain was infrequent occurring in only four events (0.32%) (Table 2, Table 3). The frequency distribution of all other AHEs attributable to vaccination were minimal (≤0.24% of the vaccination events) with most occurring as a single event. All late AHEs are summarized in Table 3 and broken down by first or second vaccination and by age group in Table 2. Based on the frequency and nature of the immediate and late AHEs reported it can be concluded that the vaccine injections were well tolerated.

**Table 3:** Frequency Distributions of Late Abnormal Health Events.

Abnormal Health Event* N = 1232	Overall N (%)	Related to IVP N (%)
Anorexia	4 (0.32%)	1 (0.08%)
Adipsia, Injection Site Abscess, Injection Site Alopecia, Injection Site Self-Trauma	0 (0%)	0 (0%)
Diarrhea	12 (0.97%)	2 (0.16%)
Emesis	8 (0.65%)	0 (0%)
Hyperthermia, Muscle Tremor, Abdominal Pain, Dehydration, Retching, Vocalization	1 (0.08%)	1 (0.08%)
Injection Site Oedema	40 (3.25%)	40 (3.25%)
Injection Site Pain	4 (0.32%)	4 (0.32%)
Lameness, Blepharitis, Burn, Central Nervous System Disorder NOS, Corneal Oedema, Corneal Ulcer, Death, Ear Infection NOS, Eye Redness, Flatulence, General Pain, Haematuria, Histiocytoma, Injection Site Reaction NOS, Murmur, Otitis Externa, Paresis, Polydipsia, Pruritus, Seroma, Skin Lesion NOS, Soft Stool, Tongue Disorder, Tooth Disorder, Urinary Bladder Disorder NOS	1 (0.08%)	0 (0%)
Lethargy	6 (0.49%)	3 (0.24%)
Alopecia Local	3 (0.24%)	0 (0%)
Cough, Urinary Incontinence, Skin Disorders NOS	2 (0.16%)	0 (0%)
Dermatitis	2 (0.16%)	1 (0.08%)

* Where specific AHEs are listed together in the table, the number of events and the percentages for each are identical.

## 4 Discussion

This report details the results of a comprehensive safety study of VANGUARD®crLyme, a vaccine for canine Lyme disease. As detailed above, immediate and late AHEs related to the administration of VANGUARD®crLyme were clinically minor and infrequent. The most frequent immediate AHE after delivery of either the first or second dose of vaccine that was attributable to vaccination was vocalization (9 events; 0.73%). Vocalization events were infrequent but slightly higher for dogs that entered the study at an age of ≤ 8 weeks age (minimum age) when compared with dogs that entered at ≥ 9 weeks of age (1.5% versus 0.36%; respectively). Potentially serious AHEs such as anaphylaxis, allergic oedema, convulsion, and urticaria (hives or red itchy welts) were not reported for any delivered vaccine dose for any dog in the study. The frequency distributions for all immediate and late AHEs assessed are detailed in Table 1, Table 2, Table 3.

The most frequently reported late AHE was injection site oedema. The total number of immediate and late injection site oedema events were 48 (3.8%). The majority of these events occurred as late stage AHEs (47/48) with just one report of injection site oedema being categorized as an immediate AHE. Of the late injection site oedema events, 28 were < 0.5″ in diameter, 18 were 0.5 to 2″ in diameter and one event was > 2″ in size. For the dogs ≤ 8 weeks of age, the mean duration of injection site oedema was 5.3 days (median 5.0 days; standard deviation 2.27 days; and minimum and maximums of 2 and 10 days). For the dogs ≥ 9 weeks of age, the mean duration of injection site oedema was 5.6 days (median 5.5 days; standard deviation 3.16 days; and minimum and maximums of 2 and 12 days). It is noteworthy that of the 47 late stage injection site oedema events, 38 were reported by a single study site (commercial breeder facility). The basis for the higher occurrence of injection site oedema at this particular site is not known but could possibly be due to vaccine delivery technique, the genetics of the purpose-bred beagles or the younger age of the dogs enrolled at the breeder facilities. Alternatively, it is also possible that the staff at the site in question were more thorough in their AHE assessments. We cannot distinguish between these possibilities. In conclusion, this study and the companion paper detailing the efficacy study of VANGUARD®crLyme demonstrated that the vaccine is efficacious and safe. The post-vaccination AHEs observed during the study were mild, transient, and associated with the normal immune response to vaccination.

## Declaration of Competing Interest

The authors declare that they have no known competing financial interests or personal relationships that could have appeared to influence the work reported in this paper.
